# Remarkable Arrest of Development

**Published:** 1883-09

**Authors:** 


### Another Use for Carbolic Acid.

Some people suffer the most intense pain and annoyance from ingrowing toe nails. If the flesh has fully embedded the edges of the nail, and the tissue has become hypertrophied about it cutting and paring seems but to aggrevate the matter. When this is the case drop a very little pure carbolic acid along the borders of the inflamed tissue, and let it soak down beneath the nail. The pain will cease as if by magic, and the irritated flesh will soon make a healthy slough. If now the nail be scraped or filed very thin in the centre only, and from that back to its root, carefully leaving the edges alone, the growth will be directed toward the middle and a complete cure will result.—*Independent Practitioner*

REMARKABLE ARREST OF DEVELOPMENT.—This case was recently exhibited before the Philadelphia County Medical Society by Dr. Atkinson. (Medical Times.) A man, aged forty, had never had teeth, nor any hair on the scalp except the downy hairs of infancy. He has no sense of smell, and lost taste. He never perspires, and when working is obliged to wet his clothes to moderate the body-heat. He can sleep in these wet clothes without catching cold. Hair is present in the axilla and on the pubis, the downy hair usually seen over the skin at large is wanting, save on the scalp. His grandmother and uncle were similarly defective. He has very good health, has never been seriously sick, and has never had dyspepsia. The secretion of urine is unusually abundant. He is married, and has eight children, among whom are two girls, both of whom lack a number of teeth.

COBWEBS are the coming remedy as a substitute for quinine, according to Spanish medical authorities. Dr. Oliva in the Correspondent Medical summarizes 119 cases of treatment by this remedy (telerana is the Spanish name for cobweb), and concludes that it will cure intermittent malarial fever, whether quotidian or tertiary. The dose is 30 grains for adults, 15 for children. It is not so immediate in its action as quinine, but more permanent, and it is tasteless. The cobwebs are selected with care, cleansed, and exposed to the sun, after which they are powdered.

A death from chloroform, administered for the purpose of tooth extraction, is reported from Scotland. A girl, seventeen years of age, a mill hand, went to Dr. Wm. Kennedy, of Penicuik, Edinburgh, to have three teeth extracted, and insisted on having chloroform administered, but with fatal result. So far as we can learn from the imperfect account of the case which has come under our notice, Dr. Kennedy seems to have been both anæsthetist and operator, though we should scarcely have expected this, now that the danger of such a practice has been so clearly shown and repeatedly commented on.

